# The Ethnopharmacological and Nutraceutical Relevance of* Launaea taraxacifolia* (Willd.) Amin ex C. Jeffrey

**DOI:** 10.1155/2018/7259146

**Published:** 2018-07-25

**Authors:** Michael Buenor Adinortey, Justice Kwabena Sarfo, Jeffery Kwarteng, Cynthia Ayefoumi Adinortey, William Ekloh, Lydia Enyonam Kuatsienu, Alexander Kwadwo Nyarko

**Affiliations:** ^1^Department of Biochemistry, School of Biological Sciences, College of Agriculture and Natural Sciences, University of Cape Coast, Cape Coast, Ghana; ^2^Department of Molecular Biology and Biotechnology, School of Biological Sciences, College of Agriculture and Natural Sciences, University of Cape Coast, Cape Coast, Ghana; ^3^West African Centre for Cell Biology of Infectious Pathogens (WACCBIP), Department of Biochemistry, Cell and Molecular Biology, College of Basic and Applied Sciences, University of Ghana, Legon, Ghana; ^4^Department of Pharmacology, School of Pharmacy, Princefield University College, Ho, Ghana; ^5^Department of Pharmacology and Toxicology, School of Pharmacy, University of Ghana, Legon, Ghana

## Abstract

*Launaea taraxacifolia* (Willd.) Amin ex C. Jeffrey is a herb found mostly in tropical Africa. The plant, commonly found in West Africa, is used in the management of many diseases including cardiovascular, respiratory, haematological, endocrine, and metabolic diseases in Ghana, Nigeria, Benin, Serra Leone, and Senegal. This piece provides comprehensive and updated information on the traditional uses, phytochemical constituents, and pharmacological and toxicological information available on* Launaea taraxacifolia* to support its medicinal uses and also unearth knowledge gaps for future studies. An electronic literature search using search engines, namely, Google Scholar, ScienceDirect, and PubMed, was carried out to obtain information on the plant. Both common and scientific names of the plant were used as keywords for the search process. This paper captured information on* Launaea taraxacifolia* from 1985 to 2018. The search revealed that the leaves of the plant possess nutritional/pharmacological effects on diseases such as diabetes mellitus, hypertension, cancer, malaria, bacterial infections, and arthritis. The leaf has been shown to be a rich source of phytoconstituents such as flavonoids, phenolic acids, tannins, alkaloids, glycosides, coumarins, triterpenoids, ascorbic acid, lycopene, and *β*-carotene. Also, isolated phytoconstituents as well as the safety profile of the plant have been documented. This review on* Launaea taraxacifolia* has provided a one-stop documentation of information in support of the several purported ethnopharmacological uses of the plant. It also reveals information gaps such as the need to research into its pharmacokinetics, interactions with drugs of importance, and its development into a plant-based drug in order to expand its clinical use.

## 1. Introduction

*Launaea taraxacifolia* (Asteraceae), also known as wild lettuce, is found mainly in tropical Africa. The plant over the years has been reported to possess many ethnopharmacological properties on disease conditions such as water retention disorders, conjunctivitis, yaws, improper bone fixation in infants, and diabetes mellitus [1–3]. The World Health Organization (WHO) has reported that between 70% and 95% of individuals in developing countries use traditional medicine also known as “complementary”, “alternative”, or “nonconventional” medicines for disease management [4]. The report added that, in Africa and the Western World, the use of traditional medicine is on the rise.

Despite the increase in patronage of traditional medicines, evidence-based scientific data on plants used as medicines are rather scattered. This emphasizes the need to collate relevant scientific and evidence-based information available to support the medicinal uses of some of these plant medicines to aid further research. Currently no topical review exists on* Launaea taraxacifolia*, thus necessitating the need to gather data available on the plant. This systematic review aimed to provide a comprehensive and updated information on the botanical aspects, ethnopharmacological uses, nutritional, phytochemistry, pharmacological activities, and toxicological assessment of extracts of* Launaea taraxacifolia* leaves to explore its therapeutic potential and evaluate future research prospects.

## 2. Method of Literature Search

**Figure 1:**
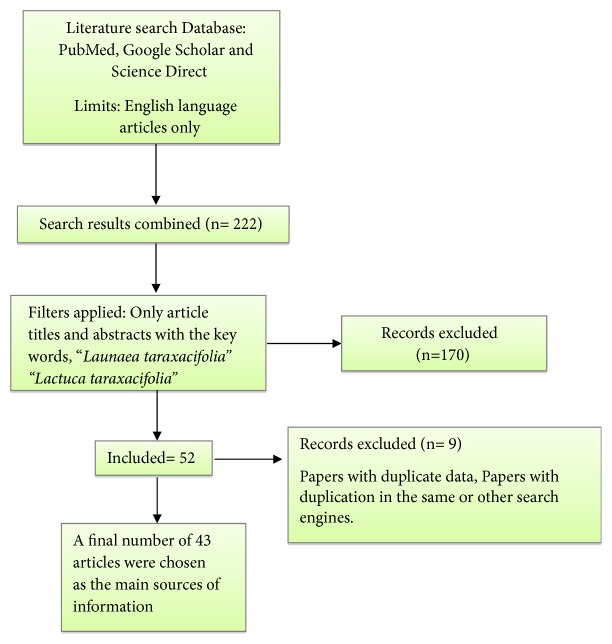
Flow diagram of systematic literature search scheme used for this review.

An online literature search was performed in January 2018, on ScienceDirect, PubMed, and Google Scholar databases using the keywords* Launaea taraxacifolia* and* Lactuca taraxacifolia* with a time limit of papers published from 1985 up to 2018 in accordance with the Preferred Reporting Items for Systematic Reviews and Meta-Analyses (PRISMA) guidelines. The search approach is illustrated in a flow diagram (Figure 1). The article selection was initially based on the following inclusion criteria: articles published in English and articles with keyword in the title, abstract or full text, and studies with isolated compounds from the plant. Articles that were retrieved were manually reviewed with the goal of identifying and excluding works that did not fit the inclusion criteria described, articles with repeated data, and duplication by the search engines.

## 3. Results

### 3.1. Taxonomy, Ethnic Names, and Ethnopharmacological Importance of Plant

*Launaea taraxacifolia* belongs to the domain Eukaryota, kingdom Plantae, and subkingdom Viridae Plantae. It is found in the Family Asteraceae or Compositae. It is of the genus* Launaea* and species* taraxacifolia* [5].  Table 1 contains various ethnic names by which the plant is called in different dialects in West Africa. Synonyms of the plant names include* Lactuca taraxacifolia* (Willd.) Schumach. ex Hornem,* Lactuca taraxacifolia* Schum. & Thonn., and* Sonchus taraxacifolius* Willd. The plant has several medicinal uses (Table 2). Its ethnomedicinal popularity has necessitated the various biological activities of the plant to be documented in numerous scientific research publications.

### 3.2. Botany and Ecological Distribution

**Figure 2:**
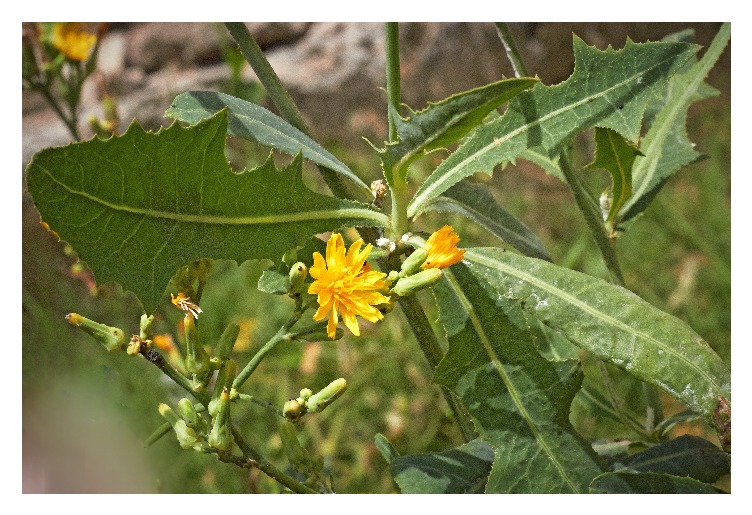
Leaves and flowers of* Launaea taraxacifolia* plant.

*Launaea taraxacifolia* is a tropical perennial plant that has a creeping root system, with its leaves at the base of an erect stem, which is about 1-3m high. The leaves are arranged in a rosette form of 3-5 and may sometimes be crowned with golden yellow corollas, which produces 7-8 nm air-borne seeds.* Launaea taraxacifolia* is unique. It is the only species in the Asteraceae family without any trichome. The leaf, stem, and nodes are potential explants for regeneration of* Launaea taraxacifolia* [6–10]. A picture of the leaves with flowers is shown in Figure 2. The plant is predominant in tropical African countries of Ghana, Nigeria, Senegal, Sierra Leone, Benin, and Tanzania with the Ethiopian highlands being known as its place of origin. It thrives at banks of water bodies, waste dumping places, and backyards [11, 12].

### 3.3. Nutraceutical and Phytochemical Composition

**Figure 3:**
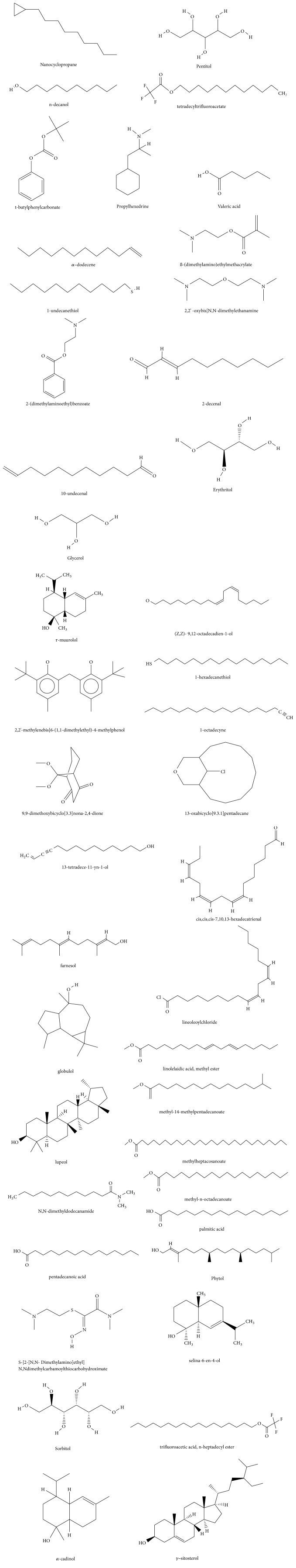
Some structures of isolated compounds from* Launaea taraxacifolia* leaves.

The leaf of the plant is consumed by indigenes of Benin, Ghana, Nigeria, Sierra Leone, and other tropical countries [11, 13–15]. It may be eaten as salads, cooked as sauces, and eaten as vegetable soup regularly during pregnancy. The plant, which forms a rich part of the African diet, is endowed with high levels of nutrients. The leaves have been reported to contain substantial amounts of macro- and micronutrients that can compare well with other conventional edible leaves. The leaves of* Launaea taraxacifolia* are endowed with saponins, terpenoids, cardiac glycosides, steroids, tannins, flavonoids, leucoanthocyanins, phenolic acids, ascorbic acid, lycopene, and *β*-carotene [16–21]. Ololade et al. [22] have isolated 47 compounds from methanolic extract of the* Launaea taraxacifolia* leaves. The most abundant chemical components found were palmitic acid, methyl-11-octadecenoate, erythritol, glycerol, linolelaidic acid methyl ester, and phytol. A long chain alcohol, 1-hexacosanol, has also been discovered to be present in the leaves [23]. Some structures of compounds isolated from the leaves of* Launaea taraxacifolia* are shown on Figure 3.

### 3.4. Pharmacological Activities

Research over the years has revealed that* Launaea taraxacifolia* possesses important pharmacological activities. These include antioxidant, hypolipidaemic, antilipid peroxidation, neuroprotective, anticancer, antimalarial, antiarthritic/anti-inflammatory, antibacteria, and cardioprotective and DNA protecting activities [24–36].  Table 3 provides an overview of reported experimental based evidence to support pharmacologic and toxicological activities of* Launaea taraxacifolia*. 

*Antioxidant Activity*. Reactive oxygen species (ROS) and Reactive Nitrogen Species (RNS) are free radicals generated by the body from normal physiological activities in body systems as well as exposure to conditions such as radiations, drugs, and pollutants. Free radicals may alter the generic structure of endogenous lipids, proteins, DNA, and other important molecules that may lead to tissue damage, triggering many diseases in that regard. The body ameliorates the effect of free radicals by synthesizing endogenous molecules, which mop up these radicals [37]. Studies have demonstrated that* Launaea taraxacifolia* leaf extract has antioxidant power to protect against oxidative stress. A study conducted by Koukoui et al. [27] highlighted that the oxidizing power of phorbol myristic acetate (PMA), an inducer of ROS such as the superoxide radical, was annulled in PLB985 cell lines by* Launaea taraxacifolia* leaf extract. Studies conducted by Ololade et al. [22], Borokini and Labunmi [26], and Adinortey et al. [24] also confirmed the antioxidant effect of* Launaea taraxacifolia* against nitric oxide (NO), 2,2-diphenyl-1-picrylhydrazyl (DPPH), and hydroxyl (OH) radicals. These activities were reported to be significant due to the high content of phenolic acids and flavonoids detected in the leaves. 

*Antilipid Peroxidation Property*. Lipid peroxidation is oxidative damage, which is normally initiated by the attack of free radicals in cellular oxidative stress. Phospholipids, lipoproteins, and other lipid-containing molecules are normally prone to peroxidation. It results in the formation of aldehydes, ketones, alkanes, carboxylic acids, and polymerization products. Malondialdehyde (MDA), an example of such reactive aldehydes, is used as a marker to determine lipid peroxidation. It forms a coloured complex with thiobarbituric acid (TBA). This principle has been used in studies conducted by Adinortey et al. [24] which demonstrated that* Launaea taraxacifolia *inhibits lipid peroxidation by 52 % with an IC_50_ value of 79 *μ*g/ml. This would imply that* Launaea taraxacifolia *could be considered as a potential agent for managing lipid peroxidation during cellular oxidative stress. 

*Hypolipidaemic Activity*. Steatosis is a clinical condition characterized by abnormal retention of lipids within a cell due to impairment of normal synthesis and degradation of fats. This abnormal lipid accumulation is often seen in individuals with obesity, hepatitis C, etc.* Launaea taraxacifolia *leaves have been documented to have hypolipidaemic effects [28]. The investigators showed that lipid accumulation induced with 0.1mM oleic acid in HepG2 cell lines was reversed when the cell lines were treated with 20 *μ*g/*μ*l of 50 % hydroethanolic extract of the plant. An* in vivo* study with Wistar rats also showed that* Launaea taraxacifolia* significantly decreased blood cholesterol and triglycerides levels significantly [28]. 

*Neuroprotective Activity*. Metals and metallic compounds interfere with functions of the central nervous [25]. Histological and biochemical studies reveal that* Launaea taraxacifolia* displays brain protective effects against lead acetate and mercuric chloride induced oxidative stress. Lead acetate and mercuric chloride caused significant (p<0.0.05) reduction of behavioural parameters such as reduction in forelimb grip strength. They also induced lipid peroxidation, reduced glutathione (GSH) level, and increased superoxide dismutase (SOD) activity. The metals also altered the microanatomy of the rat regio III cornu ammonis and cerebellum when compared with the control. These changes were significantly ameliorated (p<0.0.05) in rats cotreated with ethanolic extract of* L. taraxacifolia *when compared with the rats treated with metals only [25, 36]. 

*Anticancer Activity*. In the quest to eliminate malignant cells, contemporary medicine and therapies aimed at controlling cancerous cells have yielded undesirable side effects. The need for natural alternative medicines has thus been desirable. Anticancer effect of* L. taraxacifolia* had been purported in ethnomedicinal reports without concrete scientific data [38]. Thomford et al. [30], in an* in vitro* study, reported the anticancer effect of* L. taraxacifolia*. Their study showed that* L. taraxacifolia* significantly inhibited growth of cancerous cells by downregulating genes that control cell cycle progression of the WHC01 esophageal cancer cells. 

*Anticisplatin Damage Activity*. Cisplatin is a platinum complexed drug that is clinically used as an adjuvant of cancers and is aimed at annihilating tumor cells. Cisplatin destroys tumor cells via apoptosis or interference with transcription and/or DNA replication process. Documented side effects of this platinum complex include kidney and neuronal damage, hearing loss, and bone marrow suppression [39–41]. Aqueous extract of* L. taraxacifolia* was demonstrated to possess protective potential against cisplatin damage [31]. A study on hepatorenal histological toxicities induced by cisplatin in rats showed that* L. taraxacifolia* aqueous extracts ameliorated cisplatin-induced toxicities on liver and kidney. Dose-dependent ameliorations of these histopathologies were seen in* L. taraxacifolia* plus cisplatin-exposed groups whereas extracts-alone-treated rats had normal histoarchitecture. Hepatic (alanine aminotransferase, aspartate aminotransferase, and bilirubin) and renal (blood urea nitrogen, creatinine) parameters, which are markers of organ injury were elevated in cisplatin-exposed groups but with less severity in rats cotreated with* L. taraxacifolia* and cisplatin. Increased lipid peroxidation and decreased activities of superoxide dismutase, glutathione, and catalase were also observed in cisplatin-exposed groups. These effects were ameliorated with* L. taraxacifolia* extracts (100 mg and 400 mg). This report suggests that extract of* L. taraxacifolia* leaves reverses cisplatin-induced damage [31].

Micronuclei are markers that provide information about the integrity of chromosome structure during nuclear division. Adejuwon et al. [32] investigated the antigenotoxic potential of* Launaea taraxacifolia* (LT) aqueous leaf extract in bone marrow erythrocytes against cisplatin-induced micronuclei production in Wistar rats. Pretreatment of rats with* L. taraxacifolia* significantly reduced the increase in micronuclei polychromatic erythrocytes (MNPCEs) frequency and a concomitant decrease in polychromatic erythrocytes (PCEs) to normochromatic erythrocytes (NCEs) ratio in cisplatin-exposed groups in an extract dose-dependent fashion. The PCE/NCE ratio is an index of cytoprotection. This suggests that aqueous extract of the plant protects against cisplatin-induced damage. 

*Antimalaria Activity*.* Launaea taraxacifolia* has been reported to be used as an antimalarial agent [1, 14]. The antiplasmodial value of* L. taraxacifolia* against* Plasmodium berghei* and the chloroquine-sensitive (D6) strain* Plasmodium falciparum* was investigated. The methanolic extract of* L. taraxacifolia* leaves administered to different groups of Swiss mice at 200 mg/kg per body weight significantly (*p* < 0.05) inhibited parasitemia and improved survival time as well as some haematological parameters in* P. berghei*-infected mice. Bello et al. [34], in an* in vitro *study, also showed that the chloroform extract of* Launaea taraxacifolia* produced 52% growth inhibition to* P. falciparum* yielding an IC_50_ value of 21.55*μ*g/mL. 

*Antibacterial Activity*. The bactericidal potential of* Launaea taraxacifolia* was investigated by Ololade et al. [22] and Tayman et al. [23]. Tayman and colleagues showed that three compounds isolated from* L. taraxacifolia* significantly inhibited the growth of* Staphylococcus aureus*. Their results were confirmed by Ololade et al. who showed that, in addition to* Staphylococcus aureus*, the plant extract also inhibits* Escherichia coli, Enterococcus faecalis, Micrococcus varians, and Streptococcus agalactiae*. Implicitly there is evidence to support the fact that* Launaea taraxacifolia* is a potential inhibitor of both gram positive and gram negative bacteria. 

*DNA Protective Ability*. Electrophoresis has been used as a tool by researchers to determine distortions in native conformational structure of genetic materials such as DNA, RNA, and proteins. Adinortey and colleagues in 2018 [24] demonstrated that* Launaea taraxacifolia* leaf extract has DNA protective ability. In the study, it was observed that electrophoretic mobility was a function of DNA conformational structure. Using the UV-H_2_O_2_ model, the investigators showed that structurally damaged DNA without protection from* Launaea taraxacifolia* leaf extract had the slowest mobility on the gel compared to native supercoiled circular DNA. However, when 20mg/ml of* Launaea taraxacifolia* methanolic extract was incubated with DNA samples before UV irradiation, the electrophoretic mobility of the sample was faster and further compared to irradiated DNA sample with no extract. Cellular DNA damage had been associated with action of free radicals. UV-photolysis of H_2_O_2_ produces OH^−^ radical which is responsible for damage of genetic materials as suggested by Guha et al. [47] who had documented that OH^−^ radical binding to DNA leads to strand breakage and opening, deoxyribose sugar fragmentation, and nitrogenous base modification. The result of this study suggests that* Launaea taraxacifolia* confers protection on DNA against OH^−^ radical induced by radiation and allows less distortion in the DNA structure upon irradiation. 

*Antiarthritic and Anti-Inflammatory Activities*. Denaturation of proteins has been suggested by various researchers to be the cause of inflammation and rheumatoid arthritis. Evidence suggesting the role of ROS in rheumatoid arthritis has been on the rise. Research using Nrf2-knockout mouse has indicated that ROS are important factors in the breakdown of collagen in experimental arthritis [48–51]. Studies by Ololade et al. [22] suggested that methanolic extract of* Launaea taraxacifolia* leaves has antiarthritic/anti-inflammatory properties by preventing the denaturation of bovine serum albumin (BSA). The mechanism of denaturation involves alteration in electrostatic, hydrogen, hydrophobic, and disulphide bonding holding the globular structure of proteins in definite conformation. This activity has been associated with biologically active compounds and may be useful in drug development. 

**Figure 4:**
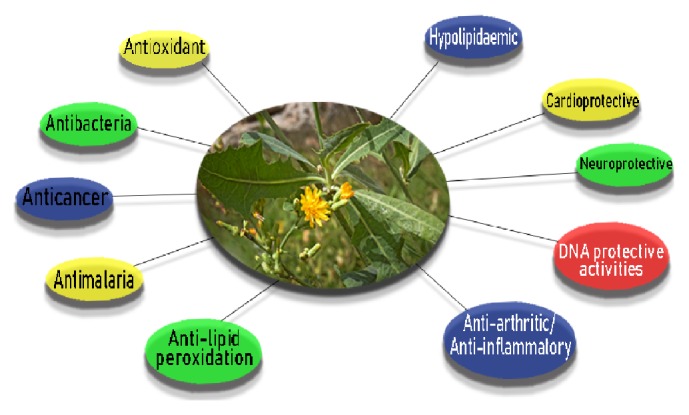
A pictorial summary of the pharmacological effects of* Launaea taraxacifolia* leaves.

*Cardioprotective Activity*. Cardiovascular diseases (CVD) are estimated to be the leading causes of mortality and morbidity throughout the world. According to the World Heart Federation [52], more than 17.1 million lives are lost each year from cardiovascular diseases. Dyslipidemia, lack of physical activities, smoking, high levels of low-density lipoprotein (LDL) cholesterol, oxidative stress, and excessive alcohol consumption are some causes of CVDs. Natural products play a significant role in drug discovery process and are useful agents for mitigating the prevalence of CVDs. Green leafy vegetables, consumed all over the world, play a vital role after maintaining the wellbeing of humans due to myriads of their phytoconstituents and nutrients [44]. The cardioprotective effect of aqueous extracts of* L. taraxacifolia* (wild lettuce) was evaluated in male Wistar rats using the isoproterenol (ISO) model of myocardial infarction. Histopathologic examination of the rats showed degeneration of myocytes with slendering of fibre, loss of nuclei, loss of cross striation of the cardiac muscles, and an increase in interfibre space (oedema). These conditions were significantly corrected in rats that were treated with* L. taraxacifolia* extract [46], thus demonstrating the protective effects of* L. taraxacifolia* extract on the cardiovascular system. A summary of the pharmacological effects of the* L. taraxacifolia* leaves is shown in Figure 4. 

*Toxicological Activities*. Toxicity studies are important in ascertaining the safety of substances to humans, especially in cases where the substances serve as food or medicines. In this regard, a number of safety studies have been conducted on* L. taraxacifolia* extracts. In an* in vivo* toxicity study by Kuatsienu et al. [29]* L. taraxacifolia* administered at a maximum dose of 1000 mg/kg per body weight of rats was found to be nontoxic in a 14-day study. They rather showed the ability of the plant to protect against gentamicin-induced kidney damage in rats. Histological studies of kidney tissues showed an insignificant change in tubular epithelium in* L. taraxacifolia* plus gentamicin treated groups compared to* L. taraxacifolia* treated only. Koukoui et al. (2017) [28] have also reported that* Launaea taraxacifolia* is nontoxic to rats at a maximum dose of 500mg/kg body weight. However, Koukoui et al. (2015) [27] have shown that the plant extract decreases HepG2 cell viability at concentrations higher than 20*μ*g/*μ*l in an* in vitro* 3-(4, 5-dimethylthiazol-2-yl)-5-(3-carboxymethoxyphenyl)-2-(4-sulfophenyl)-2H-tetrazolium (MTS) test. This is to be expected, as HepG2 cell lines are immortalized human carcinoma cells. Although, in general, there have not been negative reports from individuals who incorporate the leaves of* Launaea taraxacifolia* in their meals, there is the need, therefore, for further research to be conducted in this area to determine the most effective, yet nontoxic, dose of the plant extract.

*Launaea taraxacifolia Interactions with Drugs*. Traditional medicine is gaining prominence due to its therapeutic benefits. Some patients take orthodox drug such as antihypertensive drugs, hypoglycaemics, and antiretroviral therapy (HAART) in conjunction with traditional medicines. The combined use of traditional herbal medicines with orthodox drugs could potentially cause herb-drug interactions (HDI). These interactions may influence the bioavailability and efficacy and cause undesirable effects.

Efavirenz (EFV) and nevirapine (NVP) are main constituents of HAART and are both biotransformed by the enzyme CYP2B6 during metabolism. This drug-metabolizing enzyme may be susceptible to induction or inhibition by medicinal plant extracts. Thomford and colleagues reported in 2016 [30, 53] a study they carried out to evaluate the effect of* Launaea taraxacifolia *extract on recombinant human CYP2B6 enzyme activity on the potential of* L. taraxacifolia* extract to cause HDI and its ability to act as a reversible time-dependent inhibitor (TDI), using EVF and NVP as target drugs. The extract inhibited CYP2B6 with an IC_50_ value of 33.87 ± 1.54*μ*g/mL and a TDI of 3.17. Similar reversible time-dependent inhibitions have been reported for CYP1A2, CYP2C9, and CYP2C19. These findings show that drugs metabolized by cytochrome enzymes may be toxic to patients who use* L. taraxacifolia* concurrently with HAART. Significant levels of these drugs may accumulate in plasma thereby causing toxicity.

## 4. Conclusion

The nutritional, medicinal/pharmacological properties of* Launaea taraxacifolia *reported make it a desirable plant for development as a plant-based drug. Data presented up to date demonstrate that there is evidence that support the ethnopharmacological uses of* Launaea taraxacifolia*. The pharmacological activities reported by various researchers may be due to specific phytoconstituents. Though several compounds have been isolated from this plant, not much is known about their bioactivities. It is therefore necessary for bioactivity-guided fractionation experiment to identify specific constituents that are responsible for the desired medicinal/pharmacological effects.* In silico* molecular docking studies of the isolated compounds should be done to screen for potential inhibitors of various ailment target sites. Further studies on the structure-activity relationships of the inhibition of recombinant human cytochromes P_450_ by some bioactive compounds isolated from* Launaea taraxacifolia* leaves and their analogues are also recommended. The plant does not show any overt toxicity in* in vivo* and* in vitro* safety studies. However, there is evidence of its potential to cause herb-drug interactions through inhibition of CYP enzymes. Users should therefore exercise caution in view of potential long-term toxicity and teratogenic effects as well as harmful effects that could result from herb-herb and/or herb-drug interactions.

## Figures and Tables

**Table 1:** Ethnic names of *Launaea taraxacifolia* plant in some African countries.

People	Ethnic Group	Vernacular names	References
Ghana	Ewe (anlo)	A*ŋ*òto	[5]
	Asante Twi	Dadedru	
	Akuapem Twi	Nne noa	
	Hausa	Namijin dayii	

Benin	Awonto	Cotafon; Watchi	[13, 14]
	Holly; Yurouba	Gnanri	
	Fon; Machi	Yantotoé	
	Tchabè	Katakpa	
	Pèda; xwla	Lôto	
	Fe; Idaacha; Idasha	Ôdôdô	
	Saxwè	Wonto; Lanto	
	Adja	Wontou	

Nigeria	Yoruba	Efo yanrin	[5]
	Ibo	Ugu	

Sierra Leone	Kissi	Bekuhoa-pomboɛ	[13, 14]
	Mende	Kipo	
	Krio	ɛfo-nyori	
	Temne	a-moths*ẻ*ra	

**Table 2:** Ethnopharmacological relevance of *Launaea taraxacifolia*.

Part of plant	Reported Disease Managed	References
Leaves	Wound, sore throat, abscess	[1, 11, 14]
Leaves	Scorpion/ snake bite	[12, 14, 42]
Leaves	Anemia	[14, 43]
Leaves	Menstrual Cramps	[1, 14]
Leaves	Tetter, tinea, mycosis	[1]
Leaves	Typhoid fever	[11, 14]
Leaves	Malaria	[1, 11, 14]
Leaves	Convulsion attack	[14]
Leaves	Hypertension	[11, 13, 14, 44]
Leaves	Diabetes mellitus	[1, 11, 13–15, 44]
Leaves	Cough	[14]
Leaves	Breast milk secretion	[14]
Leaves	Indigestion, Constipation	[1, 14]
Fresh milky latex	Mosquito repellent	[11]
Fresh milky latex	Conjunctivitis	[42]
Leaves	Proper bone fixation in infants	[1, 11]
Fresh aerial part	Appetite booster	[11]
Leaves	Hepatitis	[1, 13, 44, 45]
Leaves	Bronchitis	[1]
Leaves	Mastitis	[1]
Leaves	Atherosclerosis	[1, 44]
Leaves	Chronic osteoarthritis	[1]
Leaves	Rheumatism	[1]
Leaves	Gallstones	[1]
Leaves	Pneumonia	[1]
Leaves	Cancer / tumors	[38]

**Table 3:** A summary of reported pharmacological activities of *Launaea taraxacifolia*.

Study Model	Activity	IC_50_/Effective dosage	Preparation	Reference
*In vitro*	Antioxidant	DPPH(16.18*μ*g/m) OH (128.2 *μ*g/ml) NO (123.3 *μ*g/ml))	Methanolic	[24]
*In vivo*	Antioxidant	400mg/kg bwt	Ethanolic	[25]
*In vitro*	Antioxidant	DPPH(6.59mg/ml) OH (1.94mg/ml) NO (12.8mg/ml)	Aqueous	[26]
		DPPH(3.29mg/ml) OH (2.46mg/ml) NO (4.88mg/ml)	Ethanolic	[26]
*In vitro*	Lipid peroxidation	23*μ*g/ml	Methanolic	[24]
*In vitro*	Hypolipidaemic	20 *μ*g/*μ*l	Ethanol-aqueous	[27]
*In vivo*	Hypolipidaemic	Cholesterol (500mg/kg) TAG (500mg/kg)	Ethanol-aqueous	[28]
*In vivo*	Gentamicin protective effect	300, 500mg/kg	Ethanol-aqueous	[29]
*In vitro*	HepG2 cell viability	20*μ*g/*μ*l	Ethanol-aqueous	[27]
*In vivo*	Neuroprotective	400mg/kg	Ethanolic	[25]
*In vitro*	Anti-cancer	-	Aqueous	[30]
*In vivo*	Anti-cisplatin damage	100, 400mg/kg	Aqueous	[31, 32]
*In vitro*	Anti-arthritic	0.25mg/ml	Methanolic	[22]
*In vitro*	Anti-bacteria	250,500,1000*μ*g/ml	Methanolic	[22]
*In vitro*	Antibacteria	1mg/ml	Methanol-dichloromethane	[23]
*In vitro*	DNA damage protection	20mg/ml	Methanolic	[24]
*In vivo*	Anti-malaria	200mg/kg	Methanolic	[33]
*In vitro*	Anti-malaria	21.55*μ*g/*μ*l	Chloroform	[34]
*In vivo*	Cardioprotective	200mg/kg	Aqueous	[46]
